# *Education:* An introduction to the core data for cefiderocol with reflections for a possible role within UK clinical practice

**DOI:** 10.1093/jacamr/dlab053

**Published:** 2021-06-15

**Authors:** J Andreas Karas, Jonathan Edgeworth

**Affiliations:** 1 Shionogi B.V. London, 33 Kingsway, Holborn, London WC2B 6UF, UK; 2 Department of Infectious Diseases, King's College London, London SE1 9RT, UK

## Abstract

The video in this section is a copy of a recorded webinar organized by Shionogi to launch cefiderocol to the UK market in September 2020. Dr Andreas Karas presents the key clinical data for this new antibiotic for MDR Gram-negative infections, followed by reflections from Professor Jonathan Edgeworth on the potential use of the new agent in UK clinical practice. Included in the latter is a case study example of a compassionate use case using cefiderocol. **The prescribing information for cefiderocol is available at: https://shionogi-eu-content.com/gb/fetcroja/pi.**

## Transparency declarations

This article is part of a promotional Supplement developed and sponsored by Shionogi B.V. The accompanying video formed part of an educational webinar for which the author received a speaking honorarium. The material underwent peer review by the Supplement Editors. Editorial assistance to Shionogi Europe was provided by Page Medical. J.E. received personal fees for the preparation of this article.

## Supplementary data

The video transcript is available as Supplementary data at *JAC-AMR* Online.

## Supplementary Material

dlab053_Supplementary_Data

