# Supplementary material for: Education: An introduction to the core data for cefiderocol with reflections for a possible role within UK clinical practice
Source: JAC Antimicrob Resist. 2021 Jun 15;3(Suppl 1):i17. doi: 10.1093/jacamr/dlab053 (PMC8251257; doi:10.1093/jacamr/dlab053)
Supplement: dlab053_Supplementary_Data [file dlab053_Supplementary_Data.docx]

**Supplementary data**

**Video transcript**

I am Andreas Karas; I am the Vice President of Medical Affairs at Shionogi Europe. I am also a medical microbiologist by training. And I am going to be giving you an introduction to cefiderocol and our core data.

I am going to start off by covering the structure and coverage of the molecule. Cefiderocol is a synthetic sideromycin combining a cephalosporin with a catechol siderophore. There are three important elements: the C-3 side chain, the C-7 side chain and the catechol moiety come together to form this molecule.^1^ The C-7 side chain, like the one from ceftazidime, enhances stability against β-lactamase. The C-3 side chain, similar to the one from cefepime, facilitates penetration of the Gram-negative outer membrane and confers lower affinity for β-lactamase.^1^ The catechol moiety provides the additional stability against the β-lactamases,^2^ but it is similar to the hydrolysis product of an enterobactin, and that’s what binds the free iron^3^—this makes it quite an interesting molecule.

Cefiderocol’s novel mechanism of cell entry may enable it to overcome resistance mediated by changes to porin channels and efflux pump overexpression. You can see in this diagram how cefiderocol chelates extracellular iron in one, how that chelated complex is then actively transported into the periplasm by the outer membrane receptors, where it dissociates from the iron ions and attaches to the penicillin-binding protein (PBP).^4–6^ Like other β-lactam antibiotics it can also enter the periplasm by diffusion through porins and, once inside the periplasm, it binds and inhibits PBPs – mainly PBP3.^6^

Cefiderocol is stable against all the main molecular classes of β-lactamases: A, B, C and D. The carbapenemases are in blue; in those classes are KPC, OXA-48, OXA-23/24, IMP, VIM and NDM. It is stable against all of those β-lactamases.^7^

Cefiderocol is a narrow-spectrum antibiotic; unlike most of the other drugs for treating carbapenem-resistant Gram-negative infections, it does not cover anaerobes or Gram-positives. It is the first siderophore cephalosporin. Resistance often seems to be related to that siderophore, which reduces the risk of cross-antibiotic resistance. This has high relevance for the UK because of the UK’s great variety of different resistance mechanisms seen.

I am now moving on to the topline results of some of our key studies. The first study is APEKS-NP, our pneumonia study.^8^ It was a multicentre, double-blinded, parallel group, randomized non-inferiority study with 14 day all-cause mortality as the primary endpoint. This was compared to high dose of extended infusion meropenem. The indication was patients with hospital-acquired bacterial pneumonia, ventilator-associated or healthcare-associated bacterial pneumonia (HAP, VAP or HCAP) caused by Gram-negative pathogens. There were 300 patients, with a one-to-one randomization. The slide shows the two arms of randomization, the end of treatment day 7 to 14—end of treatment plus 7 was test of cure, and the end of study was 28 days after treatment. It is important to note here that high-dose meropenem was a 2 g 3 hour infusion,^9^ given 8 hourly.

Baseline clinical characteristics: APEKS-NP included patients with augmented renal clearance as well as renal deficiency—this is an important thing to note as renal function is an important factor in the PK/PD relationship, and having augmented renal clearance, and the specified dosing for augmented renal clearance, is really important. This also shows the numbers of patients in each group, with 15% of patients in the cefiderocol arm having augmented renal clearance and 13.5% having severe renal impairment—a full spread of renal function. The APACHE II scores are also shown with around 30% of patients having a high APACHE II score across the study.^10^

Baseline Gram-negative pathogens: *Klebsiella pneumoniae*, *Pseudomonas aeruginosa* and *Acinetobacter baumannii* were the top three, with *Escherichia coli* and *Enterobacter cloacae* following. The numbers show that they are quite balanced between the arms. With regards to the type of blood culture: ‘positive’ was seen in 8.8% for cefiderocol and 10.7% for meropenem, ‘positive with Gram-negative pathogens only’ was seen in 5.4% for cefiderocol and 6.7% for meropenem, and blood cultures were not done in around 10% of patients. So, in APEKS-NP approximately 50% were Enterobacterales patients.^11–13^

The primary endpoint (Day 14 all-cause mortality^7^) for cefiderocol was 12.4%, and 11.6% for high-dose meropenem—overall, very similar primary endpoint outcomes.^7,14^ Adverse events^13^ were overall very balanced between the treatment arms, as expected—they are both β-lactams with good tolerability, as we all have experienced over the decades.

To summarize, APEKS-NP was a standard randomized clinical trial with a severely ill population and with many ITU patients. It is thus very relevant to the likely patient population where cefiderocol might be used. The comparator was chosen to be high-dose meropenem with an extended infusion, as in discussions with the regulators this was considered the gold standard at the time. We saw good efficacy against all infecting organisms, and effective treatment similar to that of meropenem.

Now moving on to the CREDIBLE-CR study. APEKS-NP was all about showing that cefiderocol works in pneumonia; CREDIBLE-CR was more about looking at the resistant pathogens in much greater detail. This was a very interesting study which was conceptualized to obtain a general understanding of the efficacy of cefiderocol, especially in those patients who have the highest unmet medical need. It had a very simple key inclusion criteria—by its nature, they enrolled patients who were very likely to be the last chance salvage therapy patients with multiple comorbidities (very similar to compassionate use cases). And unlike most antibiotic clinical trials, CREDIBLE-CR was not limited to a single infection site but included multiple sites of infections as long as there was evidence of carbapenem resistance. The important thing to note when looking at the data is that the study was not designed to provide inferential hypothesis testing, but it was intended to provide descriptive statistics of the aggregate data—so the data is presented with no formal statistical analysis, and due to limited patient numbers, you have to take care when interpreting the CREDIBLE-CR data.

Study design, it was a multicentre, open-label parallel group, randomized study which was randomized two-to-one. Cefiderocol, 2 g 8 hourly monotherapy or with one adjunctive Gram-negative antibiotic included, was compared to best available therapy (BAT) of which you could use one to three Gram-negative antibiotics, as long as they were pre-specified before randomization. There was an early assessment at Day 3, end of treatment was up to Day 21, test of cure was at end of treatment plus 7 days (the primary endpoint), then there was a follow-up and end of study 28 days after end of treatment. Again, open-label, non-inferential study with an observational BAT arm (not really a comparator arm).^7,14^

These are some of the key points regarding the background of the CREDIBLE-CR study.^7,14^ The inclusion and exclusion criteria are listed, and as I have said it was a balanced two-to-one randomization across 24 different strata, with the target pathogen not included as a stratum. Descriptive statistics showed a very heterogeneous population with three infection types: HAP/VAP/HCAP, bloodstream infection (BSI/sepsis) and UTI. Any carbapenem-resistant species was allowed, including *Acinetobacter* species and *Stenotrophomonas* species. And there were many comorbidities, including life-threatening illnesses in the patients.^7,14^

The patient disposition: 152 patients were enrolled, with the three aforementioned clinical indications shown (cUTI, BSI/sepsis and HAP/VAP/HCAP). The safety population, the micro-ITT population (patients who had a baseline Gram-negative pathogen), and the microbiological ITT population (with the carbapenem-resistant microbiology population) values are all shown, with 22, 37 and 59 in total for each arm, respectively. CREDIBLE-CR was a very challenging study to aim for; it was a carbapenem-resistant population and had very broad inclusion criteria. As I have mentioned, the population is very heterogeneous: cancer, burns, respiratory patients, multiple sites of infection. And as I have said, the study was not powered to offer statistical analysis, it was an observational trial.^7,14^

This shows the baseline drug regimens that were chosen for patients. Important things to note here are that monotherapy with cefiderocol was chosen in 82.5% of patients, and adjunctive therapy in 17.5%. This also shows a long list of different antibiotics that were chosen as adjunctive therapy: tigecycline, fosfomycin, amikacin, etc. In contrast to the BAT arm, most of the patients had combination therapy. In the colistin-based regimens, 15% got monotherapy and in the BAT arm, there were also a few patients who got monotherapy.^7,14^ In general, most of the patients in these arms had a variety of different combinations, and very few people had monotherapy—so there is quite a contrast between cefiderocol and the BAT arm.^7,14^

The primary endpoints are test of cure by infection site, and this is in the carbapenem-resistant micro-ITT population.^7,14^ Clinical cure and microbiological eradication were similar in both treatment arms, where they looked at pneumonia patients, the bloodstream/sepsis patients, or the UTI patients (who showed slightly a bigger difference).^7,14^

Now looking at one of the more interesting areas, and where the big unmet need is, which is in metallo-β-lactamase-producing organisms. Clinical cure and microbiological eradication at end of treatment, test of cure and follow-up, were where we saw some differences with cefiderocol—where clinical cure and microbiological eradication in the BAT arm was not as high as in the cefiderocol arm.^7,14^ As mentioned earlier, we have not presented any statistics as this was a non-inferential study, and the numbers seen are quite small. Nonetheless, this gives us confidence of clinical efficacy of cefiderocol in metallo-β-lactamase-producing organisms.

Efficacy conclusions^7,14^ from the CREDIBLE-CR study, are that the top three pathogens enrolled were carbapenem-resistant *A. baumannii*, *K. pneumoniae* and *P. aeruginosa*. Cefiderocol clinical cure and microbiological eradication at test of cure was consistently, numerically higher than BAT, but the study numbers are small. 82.5% of cefiderocol patients were on monotherapy, while conversely 71.1% of BAT patients were on colistin-based regimens of more than one treatment. There was a high number of indeterminates, but supplementary analysis showed no overall difference in clinical microbiology outcomes.^7,14^

Another important aspect of the study was the all-cause mortality by baseline pathogen at the end of the study. The pathogens are shown in the left column, with the cefiderocol and BAT outcomes also shown. The imbalance in the treatment groups appears mainly for some key pathogens, and mainly for *A. baumannii* where you had overall all-cause mortality at 48.7% for cefiderocol and 17.6% for BAT. Going into this higher mortality seen for *Acinetobacter* in more detail, Day 49 mortality by a pathogen for the *Acinetobacter* species was 50% compared to 24% for BAT. Looking at the patients with *P. aeruginosa* and *Acinetobacter* species, *P. aeruginosa* alone and *K. pneumoniae* alone, it can be seen that the issue of the high mortality really sits in the *Acinetobacter* patients. It is important to note here that in the APEKS-NP study, the higher *Acinetobacter* mortality was not seen—the 28 day mortality in both arms were similar.^7,14^

A key question can be posed: is this high mortality due to cefiderocol, *Acinetobacter* or patient factors? The graph on the left shows the number of isolates against each cefiderocol MIC. The white star represents each patient that had a death in the cefiderocol arm by Day 28; most of the deaths occurred in patients who had susceptible isolates—there was only one death in a patient who had an MIC of over 16 mg/L. The type of resistance mentioned is shown below the graph. With regards to patient factors, creatinine clearance, for *Acinetobacter* and other pathogens, the values are relatively balanced and slightly higher in the cefiderocol arm—the patients here had more severe renal impairment. Shock 30 days prior to randomization was higher in the cefiderocol arm (26% compared to 6%), and ICU admission at baseline for *Acinetobacter* patients was also higher in the cefiderocol arm. So, there was some imbalance in the patient stratification in the study.

Another way to look at the mortality, in the CREDIBLE-CR study is the timing of the deaths—14 day mortality, 28 day mortality and end of study mortality. Very early deaths, within less than three days of treatment, is generally a standard exclusion in studies, and there were more very early deaths in the cefiderocol arm. Late deaths, more than 28 days, are likely due to underlying illness, disease or new infection. It is important to note that only one death was considered related to BAT, as assessed by investigators at Shionogi. This is telling us that there was no difference in mortality in the Day 4 to 28, but the difference in deaths were largely later and very early in the cefiderocol arm.^7,14^ This suggests that there may be some patient factors involved.

Following those two clinical studies, the focus will now shift to SIDERO, which are *in* *vitro* studies. The *in* *vitro* activity of cefiderocol against all global SIDERO-wild-type (SIDERO-WT) isolates was a 4 year study with 38 288 isolates from various regions. This showed that 98.2% of wild-type strains were susceptible to cefiderocol, if using the EUCAST breakpoint of ≤2 mg/L.^15^ The study included large numbers of Enterobacterales, but also *Acinetobacter*, *Pseudomonas*, *Stenotrophomonas* and some *Burkholderia* as well.

Focusing now on the meropenem-resistant isolates and the *in* *vitro* activity of cefiderocol against that group, there were 4750 isolates, and 92.6% of these meropenem-resistant isolates were susceptible to cefiderocol. Again, there were substantial numbers of Enterobacterales, *Pseudomonas*, *Acinetobacter*, *Stenotrophomonas* and also some *Burkholderia* in this group.

The next section regards UK local isolate data—*in* *vitro* studies from Public Health England (PHE). For *Pseudomonas* and *Acinetobacter* isolates, some resistant isolates were identified—the mechanisms of resistance are indicated in the legend: a mixture of NDMs for *P. aeruginosa*, and the same in the *A. baumannii* isolates. This was using the PK/PD breakpoint of 2.

For UK-relevant resistance mechanisms, cefiderocol is the only antibiotic that has more than 80% susceptibility; it gets mechanisms like the KPCs and MBLs, OXA-48-like, carbapenem-resistant *P. aeruginosa* and *A. baumannii*, as well as *Stenotrophomonas maltophilia*. When looking at antibiotics in the development pipeline, those that are coming in the future—omadacycline, plazomicin, relebactam-imipenem, sulopenem and cefepime with a β-lactamase inhibitor—it is clear that cefiderocol even in this group has the highest level of *in* *vitro* activity. Looking at a bigger population outside the UK, across regions, cefiderocol again shows high rates of susceptibility against aerobic Gram-negative bacteria—in all categories: all Enterobacterales, non-fermenters, carbapenem-resistant Enterobacterales, etc. Cefiderocol provides that broad coverage for carbapenem-resistant organisms.^10,16-18^

I have shown you quite a lot of susceptibility data from reference labs and surveillance studies across the world, but how do you do susceptibility testing? At the moment we have commercially available Kirby-Bauer discs from Liofilchem® and Mastdiscs®. It is important to note that for disc testing, iron-limited agar medium is not required—you can use standard cation-adjusted Mueller–Hinton agar. We also have the availably of broth microdilution plates, which are for MICs and available from Thermo Scientific™ but labelled for ‘research use only’ at the moment. We also have our EUCAST susceptibility breakpoints for disc testing. Remember again that iron-limited medium is not required for disc testing, and you could see the zone diameter of breakpoints for Enterobacterales and *P. aeruginosa* of 22 mm, *A. baumannii* was 17 mm and *S. maltophilia* was 20 mm, and there are well-described ATCC strains for quality control for disc diffusion testing.^19^

In terms of real-world evidence, there have been compassionate use programmes running for cefiderocol across the world since 2016 because of the unmet need and the fact that many patients had pathogens that were not treatable. We have quite a few publications already, I only cover some of them, and you'll find some interesting indications and pathogens that were treated in literature.

A very interesting one from September 2020 is from Professor Falcone in Italy where cefiderocol was used as a rescue therapy for *A. baumannii* and other carbapenem-resistant Gram-negative patients in ICU. It gives us some very good insights into early use. There are also other publications on treating prosthetic joint infections, empyema and a case series from Switzerland. Look out for these compassionate use publications as they give us a very good indication of the unmet need, where patients are requiring cefiderocol, and also some early outcome data to supplement what was found in the clinical trials.

In conclusion, cefiderocol is the first siderophore cephalosporin, it is exclusively active against Gram-negative bacteria, including the WHO’s priority pathogens. It is stable against Ambler class β-lactamases, OXA-48, KPC, NDM, IMP and VIM. It has efficacy against *Pseudomonas* porin, OprD, and efflux pump resistance. I have discussed the demonstrated efficacy across clinical trials, but the CREDIBLE-CR mortality imbalance, which was discussed in depth, that is mainly associated with the *Acinetobacter* infections remains unexplained. Lastly, real-world data in WHO’s priority one critical pathogens already demonstrates efficacy. I think it is crucial for us to generate more real-world data as people start to use the product in the real-world setting.

I am now going to talk through some reflections and bring out some key points from those studies. My name is Jonathan Edgeworth, and I am a Consultant Microbiologist at Guy’s and St Thomas’ Hospital.

I think we all know that there is an urgent need for new antibiotics to treat multidrug-resistant Gram-negatives,^1,23,24^ but this is very challenging because there are lots of different species and different resistance mechanisms. These bacteria will have different epidemiological features, geographical distribution, and they affect patients in different parts of our hospitals. There are the acute patients in intensive care, and chronic patients in specialist units, such as transplantation, dialysis units and oncology. Then we often see sporadic infections sometimes admitted from the community, for either carriage or infection. So different centres will have a different experience and different issues with a variety of these organisms in different settings. I think that is what makes it so challenging to work out in the placements of new antibiotics.

Cefiderocol is a newly licensed siderophore antibiotic, of which we have heard some of the data. It has an interesting mechanism of action related to iron uptake, and its key feature is that it is stable against all four classes of β-lactamases—therefore, it has activity against all of those aforementioned organisms. This slide shows the wild-type distribution of *in* *vitro* MIC for 30 000 isolates from all four groups.^22^ It is interesting to note, I think, that there is a very broad distribution, with 3 log’s difference in MIC across these species. The breakpoint has been set at ≤2 μg/mL for the Enterobacterales and the *Pseudomonas aeruginosa*, and we are still awaiting EUCAST breakpoint for the *Acinetobacter* and *Stenotrophomonas maltophilia*.

That data was from the wild-type population, however, I think it is more likely that the first time we use cefiderocol is when we have a resistant infection and therefore the question remains about whether it is susceptible to cefiderocol, if it is resistant to other antibiotics. I think this slide gives you an indication of the likelihood that cefiderocol will be active because these are resistant carbapenem isolates that have been submitted to PHE and tested there over a 6 year period^24^. It was presented as a poster at ECCMID in 2017. On the left the species are represented, so we will see some cefiderocol MICs across most species; the red vertical line represents the breakpoint. On the right-hand side, the mechanisms of resistance in those Gram-negatives can be seen to be mostly metallo-β-lactamases, but also some variants with ESPLs and porin loss or AmpC.

If you do the same for *Pseudomonas* and *Acinetobacter*, broadly it is the same.^24^ Many resistant bacteria will be susceptible to cefiderocol, but there will be resistant isolates too—mostly the MBLs, PER and VEB in *Pseudomonas* and a mixture of OXAs in the *Acinetobacter*.^23^ This is highlighting the importance the first time you're looking to use cefiderocol, the importance of your resistant isolates, and importance of getting *in* *vitro* testing done.

Predominantly, I want to discuss the CREDIBLE-CR study. You have already seen data from the APEKS-NP study which essentially showed no difference in the primary endpoint of Day 28 mortality between high-dose meropenem, 2 g three times a day, and the cefiderocol.^9^ I think that it is a useful study to give us some general understanding of the utility of cefiderocol in an ICU population.

I would like to go into some detail about the CREDIBLE-CR study that is more challenging.^7^ I think resistant pathogen-focused studies are more challenging because, in the background of ICU with complex patients anyway, you are having to select for those with a Gram-negative pneumonia. Then on top of that, you have to predict whether they are likely to have resistant infection. You are presumably picking out a group with high end line mortality, anyway, related to the fact that they are colonized with resistant bugs and probably had a long ICU stay. The study was cefiderocol against best available therapy and, in this case, that led to 29 different regimens so it is difficult to compare cefiderocol with any one treatment; you cannot in that it is a combination of different therapies. Then it is not just one infection that you are looking at, you have three different groups in this, the nosocomial pneumonia, the sepsis and the UTI. Again, in that group, there were a high number of non-fermenters such as the *Klebsiella* species and the *Pseudomonas* that I think many of us will be used to in the UK. The high proportion of the *Acinetobacter* implies perhaps that this is a population of patients that we are perhaps less familiar with.^7,14^

The clinical cure and microbiological eradication were the same in the cefiderocol and the best available therapy group.^7^ Although, I think it is fair to say that it was comparatively low in both, with approximately 40%–50% clinical cure and approximately 20%–30% microbiological eradication.^7^ Again, I think that this is probably related to the underlying complexity of these patients and the challenges of actually eradicating Gram-negatives when these patients are colonized because they are intubated with multiple comorbidities.

An important feature of the CREDIBLE-CR study was that the end of study mortality was higher in the cefiderocol arm at 34% compared to 18%.^7,14^ It is going to be important to work out why that is and that is not something we are going to explain today. I just want to drive a couple of features home—a couple of observations. This mortality was predominantly in the *Acinetobacter* population. It was 50% in the cefiderocol arm and 24% in the best available therapy arm. There is also this higher mortality observed when *Acinetobacter* was coinfecting with *Pseudomonas*, but when you looked at the *Pseudomonas* and the *Klebsiella* alone, the mortality in the two arms was the same.^7,14^

What is different about the *Acinetobacter* infections? I think that is the question we have to ask. When you look back at the APEKS-NP study, the Day 28 mortality in the cefiderocol and the meropenem arm was the same so it was not something we had seen before. The question will be, is this high mortality due to the antibiotic effect? Is it down to something specific with this antibiotic, or is it the patient?

I have two observations to make today, that it does not appear to be due to the MIC of the *Acinetobacter*. Here you can see the majority of the patients who died in the cefiderocol arm, marked with an asterisk, have low MIC. However, when you look under the patient characteristics, of those randomized to the *Acinetobacter* arm, there was a higher prevalence of shock and presence in ICU at baseline in the cefiderocol arm, presumably by chance. So, it is possible that it is those underlying factors that we know play a significant role in the outcome of the patients, that impacted on their outcome in ICU. While we are waiting for more detail on those patients from that study for us to really get an understanding of what went on, I think it highlights the importance of getting some useful information from looking at real-world cases^28^—the experience of people when they first use cefiderocol. I think this is a good study here that has run through the details of seven examples where cefiderocol has been used. These are complex patients with multiple resistant organisms and often they are treated with cefiderocol plus other antibiotics for a long time.^25^ It is worth looking at those examples.

The last thing I would like to do now is just to present the experience we had with a patient that we treated with cefiderocol to illustrate some of the challenges, and also point out some of the useful observations that you can get through real-world use. This was a patient who was an international transfer from Kuwait. When they arrived, they were colonized and infected with multiple different Gram-negatives just like the examples that I showed in that case review. *Pseudomonas aeruginosa* was in the blood and on the catheter tip that was removed on admission. Another catheter tip had a carbapenem-resistant *Acinetobacter*, and the rectal screen had a carbapenem-resistant *Klebsiella*.^26^ So, all three in one go on the same patient. They were with us for a while. I'm just going show a couple of slides showing what happened during that time course.

This is broadly the first 4 weeks during which there was persistently *Pseudomonas* in the blood cultures, only sensitive to colistin and gentamicin. We started with colistin and gentamicin but when the bacteria was growing through that, we changed the gentamicin to meropenem. Surprisingly, the MIC *Pseudomonas* with meropenem was greater than 32, the blood cultures cleared, and the patient got better. We did an echocardiogram (echo) on admission and it showed a thickened aortic valve, but there was no obvious aortic regurgitation. Around Week 3, the patient became septic again and blood cultures again became positive for *Pseudomonas*. We restarted initially on the colistin and the gentamicin and that did not help change the blood. We then again swapped the gentamicin for meropenem and the blood cultures were negative. When we repeated the echo, this time there was reported possible vegetation with some mild to moderate aortic regurgitation. This was actually strongly suspicious at that point that the patient had endocarditis.^26^

This shows the subsequent time course of the patient with us, and you can see the blood cultures did go negative initially on the colistin and the meropenem. We suspected that that would not affect a medical cure and the patient’s access for valve surgery. They were quite unwell at the time so that assessment was prolonged longer than we would have liked. During that time, the blood cultures became positive again; the colistin and meropenem had failed, and that led to a further delay while we sought alternative agents. There was nothing that we could identify through *in* *vitro* testing, so we contacted Shionogi. Within a 2 week period, we were able to get hold of the cefiderocol, going through the government processes, and we started that at around Day 85.^26^ We gave six doses of cefiderocol before they went for surgery. We took a blood culture before surgery, then the patient had a successful aortic valve replacement. The blood culture that was taken before surgery was negative, as were all subsequent blood cultures. The valve did not grow. We did a PCR of it to ensure that the material we were actually culturing had evidence of presence of the organisms. It was PCR positive for *Pseudomonas*, but it did not grow. We felt that six doses of cefiderocol, blood cultures negative after being positive for weeks, indicated some sort of microbiological activity in the cefiderocol. We were also quite surprised that the valve did not grow as well after such a short period of time. Although the patient’s improvement, subsequently leaving hospital and going back to Kuwait, was due to, in large, the aortic valve surgery, we took from this case some insight into the cefiderocol having some action and activity in a very serious and difficult-to-treat sterile site infection.^26^

Obviously given the organisms in which we are likely to be first using cefiderocol, it is important to do susceptibility testing,^27–29^ and you need to have that on site. There are these broth microdilution plates, in which you can include cefiderocol alongside other antibiotics. Also, disc testing; zone sizes have been set and interestingly, the *Pseudomonas* that we tested, all those bloods to infection isolates, some of them were just below the breakpoints in zone diameter and some were above.^19^ We spent a lot of time doing this *in* *vitro* testing and still were uncertain about the efficacy of the cefiderocol because it was around the breakpoint, but it has still had a good effect. I think it is important to keep that in mind when you do disc testing.

I think it is fair to say we all realize that there is a need for new antibiotics, and cefiderocol is a good option that is now available and licensed. It has this *in* *vitro* activity against all of these representatives of these carbapenem-resistant organisms when we see them. There are randomized controlled studies supporting the licensing, which give us confidence in its safety and appropriateness in a number of different settings.

The CREDIBLE-CR study was a challenging study with recruiting resistant organisms and comparing cefiderocol against a variety of different combinations. Although the clinical microbiological endpoints were met, we did see this increased all-cause mortality in the *Acinetobacter* infections that is not due to the *in* *vitro* susceptibility. Clearly that needs to be investigated further to work out what it was about. In the meantime, it is helpful to gain one’s own experience and also to review real-world experience, such as the case I showed of endocarditis, that give us insight into where cefiderocol may be helpful.

The video of this presentation includes the prescribing information for Fetcroja (cefiderocol).

References

**1**. Sato T, Yamawaki K. Cefiderocol: discovery, chemistry, and *in vivo* profiles of a novel siderophore cephalosporin. *Clin Infect Dis* 2019; **69** Suppl 7: S538–42.

**2**. Ito-Horiyama T, Ishii Y, Ito A *et al.* Stability of novel siderophore cephalosporin S-649266 against clinically relevant carbapenemases. *Antimicrob Agents Chemother* 2016; **60**: 4384–6.

**3**. Ito A, Kohira N, Bouchillon SK *et al. In vitro* antimicrobial activity of S-649266, a catechol-substituted siderophore cephalosporin, when tested against non-fermenting Gram-negative bacteria. *J Antimicrob Chemother* 2016; **71**: 670–7.

**4**. Ito A, Nishikawa T, Matsumoto S *et al.* Siderophore cephalosporin cefiderocol utilizes ferric iron transporter systems for antibacterial activity against *Pseudomonas aeruginosa*. *Antimicrob Agents Chemother* 2016; **60**: 7396–401.

**5**. Ito A *et al. ASM Microbe, New Orleans, LA, USA, 2017*. Poster Saturday-114.

**6**. Zhanel GG, Golden AR, Zelenitsky S *et al.* Cefiderocol: a siderophore cephalosporin with activity against carbapenem-resistant and multidrug-resistant Gram-negative bacilli. *Drugs* 2019; **79**: 271–89.

**7**. Shionogi. Antimicrobial Drugs Advisory Committee Cefiderocol Briefing Document. NDA #209445, October 2019.

**8**. ClinicalTrials.gov. Clinical Study of Cefiderocol (S-649266) for the Treatment of Nosocomial Pneumonia Caused by Gram-negative Pathogens (APEKS-NP). <https://clinicaltrials.gov/ct2/show/record/NCT03032380>.

**9**. Wunderink, Matsunaga Y, Ari M *et al.* LB4. Efficacy and safety of cefiderocol vs. high-dose meropenem in patients with nosocomial pneumonia—results of a Phase 3, randomized, multicenter, double-blind, non-inferiority study. *Open Forum Infect Dis* 2019; **6** Suppl 2: S994.

**10**. EMA. Fetcroja European Public Assessment Report, Apr 2020. <https://www.ema.europa.eu/en/documents/assessment-report/fetcroja-epar-public-assessment-report_en.pdf>.

**11**. Torres A, Zhong N, Pachl J *et al.* Ceftazidime-avibactam versus meropenem in nosocomial pneumonia, including ventilator-associated pneumonia (REPROVE): a randomised, double-blind, phase 3 non-inferiority trial. *Lancet Infect Dis* 2018; **18**: 285–95.

**12**. Kollef MH, Nováček M, Kivistik Ü *et al.* Ceftolozane-tazobactam versus meropenem for treatment of nosocomial pneumonia (ASPECT-NP): a randomised, controlled, double-blind, phase 3, non-inferiority trial. *Lancet Infect Dis* 2019; **19**: 1299–1311.

**13**. Wunderink RG, Giamarellos-Bourboulis EJ, Rahav G *et al.* Effect and safety of meropenem-vaborbactam versus best-available therapy in patients with carbapenem-resistant Enterobacteriaceae infections: the TANGO II randomized clinical trial. *Infect Dis Ther* 2018; **7**: 439–55. (Supplementary eTable 4).

**14**. Bassetti M, Echols R, Matsunaga Y, *et al*. Efficacy and safety of cefiderocol or best available therapy for the treatment of serious infections caused by carbapenem-resistant Gram-negative bacteria (CREDIBLE-CR): a randomised, open-label, multicentre, pathogen-focused, descriptive, phase 3 trial. *Lancet Infect Dis* 2021; **21**: 226–40. <https://doi.org/10.1016/S1473-3099(20)30828-8>.

**15**. Shionogi data on file.

**16**. Tacconelli E, Carrara E, Savoldi A *et al*. Discovery, research, and development of new antibiotics: the WHO priority list of antibiotic-resistant bacteria and tuberculosis. *Lancet Infect Dis* 2018; **18**: 318–27.

**17**. Wu JY, Srinivas P, Pogue JM. Cefiderocol: a novel agent for the management of multidrug-resistant Gram-negative organisms. *Infect Dis Ther* 2020; **9**: 17–40.

**18**. EUCAST. Cefiderocol: Rationale for EUCAST Clinical Breakpoints. Updated CHMP, Apr 2020.

**19**. EUCAST. Breakpoint Table for Interpretation of MICs and Zone Diameters, Version 10.0, Cefiderocol Addendum April 2020. [https://www.eucast.org/clinical_breakpoints](https://www.eucast.org/clinical_breakpoints/).

**20**. Hammoudi Halat D, Ayoub Moubareck C. The current burden of carbapenemases: review of significant properties and dissemination among Gram-negative bacteria. *Antibiotics* 2020; **9**: 186.

**21**. Shionogi. Fetcroja (cefiderocol) Summary of Product Characteristics.

**22**. Yamano. *In Vitro* Activity of Cefiderocol Against a Broad Range of Clinically Important Gram-negative Bacteria. Cefiderocol Surveillance Studies. Shionogi, Pharmaceutical Research Division.

**23**. Mushtaq S, Vickers A, Hussain A *et al. In-vitro* activity of cefiderocol (S-649266) against multidrug-resistant Enterobacteriaceae from the UK. *Twenty-seventh European Congress of Clinical Microbiology and Infectious Diseases, Vienna, Austria, 2017.* Poster P1315.

**24**. Mushtaq S, Sadouki Z, Vickers A *et al. In vitro* activity of cefiderocol against extensively drug-resistant *Pseudomonas aeruginosa* and *Acinetobacter baumannii* from the UK. *Twenty-ninth European Congress of Clinical Microbiology and Infectious Diseases, Amsterdam, The Netherlands, 2019.* Poster P1860.

**25**. Zingg S, Nicoletti GJ, Kuster S *et al*. Cefiderocol for extensively drug-resistant Gram-negative bacterial infections: real-world experience from a case series and review of the literature. *Open Forum Infect Dis* 2020; **7**: ofaa185.

**26**. Edgeworth JD, Merante D, Patel S *et al.* Compassionate use of cefiderocol as adjunctive treatment of native aortic valve endocarditis due to extremely drug-resistant *Pseudomonas aeruginosa*. *Clin Infect Dis* 2019; **68**: 1932–4. Speaker’s own.

**27**. Liofilchem®. Antibiotic Discs. <https://www.liofilchem.com/en/products/liofilchem-products/category.html?classe=020>.

**28**. The Mast Group Ltd. Cefiderocol 30 μg Cartridge Discs. <https://www.mast-group.com/uk/products/ast/antibiotic-susceptibility-test-discs-in-cartridges/fdc30c/>.

**29**. Thermo Fisher Scientific. Sensititre™ Custom Plate: RUO MIC or BP Not Including Yeast or Mycobacterium. <https://www.thermofisher.com/order/catalog/product/DRIEDPLATE?sid=SRCH=srp-DRIEDPLATE#/DRIEDPLATE?SID=srch-srp-DRIEDPLATE>.
